# Computational identification of the normal and perturbed genetic networks involved in myeloid differentiation and acute promyelocytic leukemia

**DOI:** 10.1186/gb-2008-9-2-r38

**Published:** 2008-02-21

**Authors:** Li Wei Chang, Jacqueline E Payton, Wenlin Yuan, Timothy J Ley, Rakesh Nagarajan, Gary D Stormo

**Affiliations:** 1 Department of Biomedical Engineering, Washington University, St Louis, MO 63130, USA; 2 Department of Pathology and Immunology, Division of Laboratory Medicine, Washington University School of Medicine, St Louis, MO 63110, USA; 3 Division of Oncology, Department of Medicine, Washington University School of Medicine, St Louis, MO 63110, USA; 4 Department of Genetics, Washington University School of Medicine, St Louis, MO 63110, USA

## Abstract

**Background:**

Acute myeloid leukemia (AML) comprises a group of diseases characterized by the abnormal development of malignant myeloid cells. Recent studies have demonstrated an important role for aberrant transcriptional regulation in AML pathophysiology. Although several transcription factors (TFs) involved in myeloid development and leukemia have been studied extensively and independently, how these TFs coordinate with others and how their dysregulation perturbs the genetic circuitry underlying myeloid differentiation is not yet known. We propose an integrated approach for mammalian genetic network construction by combining the analysis of gene expression profiling data and the identification of TF binding sites.

**Results:**

We utilized our approach to construct the genetic circuitries operating in normal myeloid differentiation versus acute promyelocytic leukemia (APL), a subtype of AML. In the normal and disease networks, we found that multiple transcriptional regulatory cascades converge on the TFs Rora and Rxra, respectively. Furthermore, the TFs dysregulated in APL participate in a common regulatory pathway and may perturb the normal network through Fos. Finally, a model of APL pathogenesis is proposed in which the chimeric TF PML-RARα activates the dysregulation in APL through six mediator TFs.

**Conclusion:**

This report demonstrates the utility of our approach to construct mammalian genetic networks, and to obtain new insights regarding regulatory circuitries operating in complex diseases in humans.

## Abstract 2

A dissection of the genetic networks and circuitries is described for two form of leukaemia. Integrating transcription factor binding and gene expression profiling, networks are revealed that underly this important human disease.

## Background

Acute myeloid leukemia (AML) comprises a group of diseases characterized by abnormal myeloid differentiation and an accumulation of abnormal myeloid cells in the bone marrow and peripheral blood. Like other complex diseases in humans, AML is likely to be caused by disruption or dysregulation of multiple regulatory pathways. Recent studies have demonstrated a key role for aberrant transcriptional regulation in AML pathophysiology. Namely, many lineage-specific transcription factors (TFs), which coordinate normal myeloid development, are often mutated or altered in genetic fusions produced by chromosomal translocations [1,2]. Moreover, participants of many of these chimeric proteins are themselves TFs [3,4]. These TFs may in turn interact with the normal genetic circuitry involved in myeloid differentiation and induce downstream events in AML pathogenesis. Although several chromosomal fusion proteins and myeloid TFs involved in leukemia have been identified and studied independently, how each individual TF interacts with others, and how each regulatory pathway correlates with others, remains unclear. Such comprehensive delineation of the genetic networks underlying both normal myeloid differentiation and leukemia is crucial to better understand AML pathophysiology and to develop improved therapeutic strategies.

Uncovering genetic networks has been a great challenge in the post-genomic era. Breakthroughs in experimental methods such as chromatin immunoprecipitation followed by promoter arrays [5] have vastly improved the efficiency of TF target identification [6,7], but these methods may be applied to only one TF under one condition in one experiment and, therefore, are laborious and time consuming. Alternatively, computational methods seek to solve this problem using a systems biology approach. A majority of these methods have utilized analysis of gene expression profiling experiment data to construct a coexpression network. These approaches usually apply computational algorithms or machine learning techniques such as analytical methods [8,9], statistical regression [10], Bayesian networks [11-13], support vector machine [14], data processing inequality [15] and minimum description length principle [16]. However, due to the complexity of expression data (that is, the expression of many genes are measured only at a few data points), it is generally difficult to identify the dependencies and interactions between TFs and their target genes accurately. One common challenge of expression profiling based methods is to distinguish coregulation from coexpression. Namely, genes that are coherently expressed with a TF are not necessarily directly regulated by that TF. Therefore, most of these methods have focused on simpler organisms, such as bacteria or yeast, in which the number of TF genes is small and the structure of the regulatory network is simpler.

Another approach to constructing genetic networks is based on identification of TF binding sites. This approach either predicts the transcriptional regulators of a set of coexpressed genes [17,18] or predicts the regulatory targets of TFs using their binding sites [19,20]. In these methods, a model of TF binding elements is first built by experimental or computational methods. This model is then used to search for genes that have matching sites in their non-coding sequence. A network of transcriptional regulation may be constructed by identifying targets for each individual TF. The advantage of this approach over expression based methods is that it identifies direct regulatory targets of a TF. However, its performance is strongly based on the accuracy of the TF binding site identification. Due to the high false discovery rate of TF binding sites, this approach has primarily been successful in simpler organisms [17,21], and applying this approach to mammals is still difficult and challenging.

Because each of these approaches has its own advantages and limitations, recent studies have taken an integrated approach to combine multiple types of information in order to make better predictions on regulatory networks. These methods include combining gene expression data with TF binding site analysis [22-24], combining chromatin immunoprecipitation with gene expression data [25-27], and combining chromatin immunoprecipitation data with regulatory motif discovery [28]. Although the performance of these integrated approaches is superior compared to the individual methods, most of them have been designed and tested only in lower eukaryotes. Therefore, the accurate identification of genetic networks in mammals remains a challenging problem.

In this report, we present a novel approach to inferring genetic networks in mammals by combining gene expression profiling data and TF binding site analysis. We utilize this approach to study the genetic networks operating in myeloid differentiation and to elucidate how this circuitry goes awry in acute promyelocytic leukemia (APL), a subtype of AML. APL was chosen because its pathogenesis is likely based on a common mechanism involving transcriptional dysregulation. Namely, APL is characterized by the presence of a chromosomal fusion protein, PML-RARα [4]. One participant of this chimeric protein, RARα, is a TF. Therefore, it is feasible that disruption of RARα function initiates the dysregulatory events in APL and is thus a good model for predicting the perturbation of genetic networks. Using our analytical approaches, we first constructed the genetic network underlying normal myeloid differentiation. In this network, multiple transcriptional regulatory cascades converge on Rora, indicating a novel function in modulating myeloid development. Next, using expression data in APL, we identified a set of dysregulated TFs and predicted their aberrantly expressed targets. These dysregulated TFs formed a genetic pathway distinct from the normal network that converged on Rxra and interacted with the normal network through Fos. Finally, we identified a set of direct targets for PML-RARα and proposed a role for this set in APL pathogenesis. Together, these results provide novel insights regarding the genetic circuitry underlying myeloid differentiation and APL pathophysiology, and our analytical approach demonstrates the utility of an integrated strategy for genetic network construction that may be applied to study other complex diseases in humans.

## Results

### Construction of transcriptional regulatory networks

**Figure 1:**
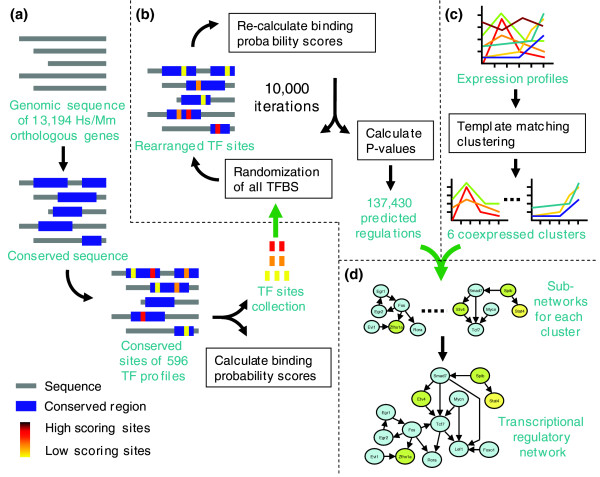
Workflow of genetic networks construction. This workflow contains four major stages. **(a) **TF binding site identification. Genomic sequences of annotated genes are retrieved and aligned, and conserved TF binding sites in genomic sequences are identified. Binding probability scores are calculated using the identified binding sites. **(b) **TF target identification. The *P *value for observing a given binding probability score or higher by chance is calculated using permutation of TF binding sites. Using a *P *value cutoff, regulatory targets of each TF are identified. **(c) **Coexpressed gene cluster identification. Gene expression profiles are collected from experiments. Coherently expressed genes are identified and clustered. **(d) **Network construction. Genetic networks are identified for each coexpressed gene cluster using the target genes predicted for each TF within each gene cluster. The complete regulatory network is then constructed by consolidating individual networks. Hs, *Homo sapiens*; Mm, *Mus musculus*.

Our strategy to identify transcriptional regulatory networks combines two independent, but complementary methods: TF binding site identification and analysis of gene expression profiling data (Figure 1). TF binding site analysis is used to identify genes containing overrepresented binding sites of a TF, whereas analysis of gene expression profiling data results in one or more genes that are coherently expressed with a TF. Our hypothesis is that genes identified using both methods are more likely to be regulated by a TF than those genes identified by either of the methods alone. Thus, to predict regulatory targets of a TF by sequence analysis, all the TF binding sites that are conserved in human and mouse were identified, and binding probability scores of each TF binding each gene were calculated using a regulatory sequence analysis pipeline [29] (Figure 1a). To identify genes with a statistically significant binding score, the *P *value for observing a binding score by chance was calculated by randomizing all the identified TF binding sites in the genome (Figure 1b). In parallel, gene expression profiles were obtained from cultured bone marrow cells that are stimulated with the granulocyte colony-stimulating factor (G-CSF) growth factor to simulate the *in vivo *myeloid differentiation program [30]. This system allowed us to identify important genes that are upregulated during myeloid differentiation. Template matching was used to identify genes whose expression is similar to a specific expression pattern or 'template'. This step identified six coexpressed gene clusters (Figure 1c). The regulatory interactions identified by sequence analysis were used to construct regulatory networks for each cluster, which were then consolidated to establish the regulatory network underlying myeloid differentiation (Figure 1d). Each of these steps is described in greater detail in the following sections.

### Transcription factor target identification by regulatory sequence analysis

To identify regulatory targets of TFs using genomic sequence information, all of the evolutionarily conserved TF binding sites in the human genome were identified using 596 known TF binding profiles curated in the TRANSFAC [31] and JASPAR [32] databases. Using TF binding sites found in the non-coding sequence of a gene, binding probability scores [29], which assess the likelihood of a TF regulating a gene, were calculated for each TF-gene pair (Figure 1a). The *P *value for observing a binding score for a TF-gene pair by chance was then calculated by permutation of all the binding sites in the genome (Figure 1b). By applying a *P *value cutoff, genes that have statistically significant binding scores for a TF were identified as putative targets of that TF. The appropriate *P *value cutoff was determined empirically to be 0.005 by using the total number of transcriptional regulatory interactions estimated in a previous study [15]. As a result, 106,997 TF-target gene pairs (that is, a TF regulating a target gene), including 6,474 TF-target TF pairs (that is, a TF regulating another TF gene) were identified. Using human-mouse ortholog gene pairs calculated using the HomoloGene database (see Materials and methods), these transcriptional regulatory interactions predicted in human were mapped to orthologs in mouse and thus generated 102,346 TF-target gene pairs. To determine if these regulatory relationships were supported by other computational prediction methods, these results were compared to the data curated in PReMod, a database of genome-wide *cis*-regulatory module predictions [33]. As a result, 40.3% of these TF-target pairs were also predicted in PReMod.

### Identification of upregulated gene clusters during myeloid differentiation

**Figure 2:**
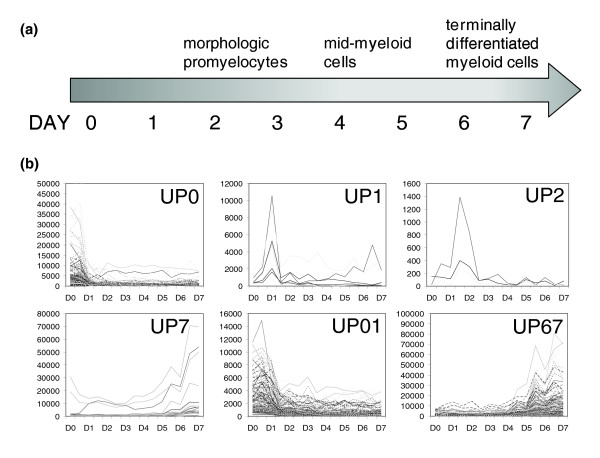
Coexpressed gene clusters identified during myeloid development. **(a) **The predominant cells in culture during the seven-day myeloid differentiation time course are promyelocytes, mid-myeloid cells, and terminally differentiated myeloid cells cultured at days 2 and 3, days 4 and 5, and days 6 and 7, respectively. **(b) **Coherently expressed gene clusters were identified for genes upregulated on just one day (UP0, UP1, UP2 and UP7) or over two consecutive days (UP01 and UP67) during *in vitro *myeloid differentiation.

To elucidate the transcriptional regulatory networks underlying myeloid development, expression profiling data were utilized from a previous study that employed a well characterized model of *in vitro *myeloid differentiation [30]. In this model, G-CSF is used to stimulate the maturation of enriched myeloid progenitors. During the seven-day time course, the predominant cells in culture at days 2 and 3, at days 4 and 5, and at days 6 and 7 are promyelocytes, mid-myeloid cells, and terminally differentiated myeloid cells, respectively (Figure 2a). Using these data, we identified coherently expressed genes during myeloid differentiation. Because myeloid development is a unidirectional, progressive event, it was hypothesized that genes regulated during this process have relatively simple expression patterns (that is, up-regulated or down-regulated at one or more points during myeloid development). In fact, comparing gene expression profiles during the *in vitro *system to an exhaustive list of temporal patterns revealed that the majority of genes that were triggered at some point in the seven-day time course were up-regulated either on just one day or over two consecutive days (Additional data file 1). Therefore, we focused on these two types of expression patterns. Expression patterns that were upregulated on just one day (day 0 to day 7) or over two consecutive days (days 0 and 1 to days 6 and 7) during the myeloid development were defined as 'templates'. The Pearson's correlation coefficient was calculated for each gene expression profile and template. To focus on the transcriptional regulation of the most coherently expressed genes, a correlation coefficient cutoff of 0.9 was used to identify genes whose expression profiles match each template. Using this method, six coexpressed gene clusters were identified, including genes upregulated on day 0, 1, 2, or 7, and genes upregulated on days 0 and 1 or days 6 and 7 (Figure 2b; Additional data file 2). The clusters that were upregulated on day 0, on days 0 and 1, and on days 6 and 7 include the most genes (267, 138, and 118 genes, respectively). These clusters contain many well known genes that are associated with myeloid differentiation, including those encoding myeloid differentiation antigens (for example, *Cd2*, *Cd3d*, *Cd5*), and terminal myeloid differentiation genes (for example, *Mmp9*, *Fpr1*, and *Itgam*).

### Integration of sequence analysis and expression profiling to construct genetic networks

**Figure 3:**
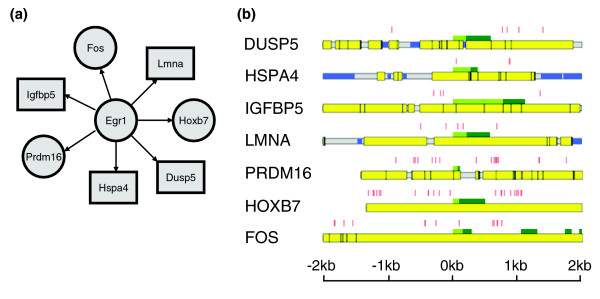
Predicted regulatory targets of Egr1 in myeloid differentiation. **(a) **Seven genes were identified as direct regulatory targets of Egr1. Three of these genes encode TFs (circle nodes). **(b) **Evolutionarily conserved Egr1 binding sites (red bars) were identified in the ± 2 kb proximal promoter region of the predicted target genes. All the Egr1 binding sites were conserved in human, mouse and rat except for PRDM16, whose rat ortholog was not available. Gene annotation information is color coded: blue, repetitive elements; yellow, conserved sequence; dark green, coding region; light green, untranslated region.

The results of genomic sequence analysis and expression profiling analysis were integrated to construct the genetic network associated with each coexpressed gene cluster. The regulatory targets of myeloid TFs were identified by the intersection of genes found in the same cluster with a TF (that is, those having a similar expression pattern to that of the TF) and genes having statistically significant binding scores with that TF. Using this approach, 96 and 25 TF-target gene pairs were identified for the gene clusters upregulated on day 0 and days 0 and 1, respectively (Additional data file 3). Note that there were also genes that were down-regulated on days 0 and 1 and days 6 and 7 (Additional data file 4), but none of the TFs that were up-regulated at those time points and that had a known binding profile were predicted as a regulator of these genes. Thus, these genes may be regulated by other myeloid TFs whose binding profiles are not yet available. Using the entire set of predicted TF-target gene pairs, a genetic network was constructed for each gene cluster (Additional data file 5). In these networks, TFs and their target genes are represented by nodes, and a directed edge is drawn from a TF to a gene if the TF regulates the gene. The identified genetic networks allowed for the identification of previously unknown TFs that regulate myeloid differentiation as well as regulatory target genes of known myeloid regulators. For example, Egr1 was shown to be a candidate myeloid regulator by previous studies [34-36]. However, what myeloid genes are directly regulated by Egr1 is unclear. Using our results, seven genes were found to be potentially regulated by Egr1, including three genes encoding TFs (Figure 3a). Among these genes, *Dusp5 *and *Egr1 *are both strongly upregulated after interleukin-5 treatment in eosinophils [37]. *Lmna *modulates cellular responses to the transforming growth factor-beta 1 (*Tgfb1*) signaling pathway [38], and *Tgfb1 *is regulated by Egr1 [39]. Further computational analysis showed that all of these genes have evolutionarily conserved Egr1 binding sites in their proximal promoter region (Figure 3b). These results suggest that the regulatory networks constructed by our method identified potential target genes of Egr1.

### Expanding genetic networks by additional myeloid transcription factors

The previous analysis identified myeloid TFs that are coherently expressed with their target genes during myeloid differentiation. However, there may be additional myeloid TFs that may not share a similar expression profile with their target genes (for example, myeloid TFs that are constantly expressed). The TF binding site analysis described above identifies TFs that regulate individual genes in the coexpressed gene clusters. Thus, PAP [29] was utilized to identify additional myeloid TFs that regulate a set of genes in myeloid gene clusters. PAP scores each TF and predicts TFs that regulate a set of coexpressed genes using a statistical model that is based on TF binding sites and that is used to calculate a *P *value to assess the statistical significance of this binding (see Materials and methods). Using a *P *value cutoff of 0.05, up to five additional TFs were identified for each of the coherently expressed gene clusters (Table 1). A majority of these additional TFs are known myeloid regulators or are involved in leukemia pathophysiology, including AML1, PU.1, and C/EBPα. These TFs were added into the genetic network for each gene cluster as new nodes (Additional data file 6), and connections from these TFs to other genes in each individual network were made based on the TF-target gene pairs predicted by genomic sequence analysis (Additional data file 7).

**Table 1:** Additional myeloid transcription factors identified by PAP

Cluster	Accession	TF	Symbol	Association with myeloid development	Reference
UP0	MA0081	SPI-B	*Spib*	Can functionally replace PU.1 in myeloid development	[85]
	M00961	VDR	*Vdr*	Involved in monocytic differentiation in human leukemia cells	[86]
	M00777	STAT	*Stat4*	Expressed in early myeloid development	[79]
	MA0103	deltaEF1	*Zfhz1a*		
	M00655	PEA3	*Etv4*		
UP1	M00161	Oct-1	*Pou2f1*	Regulates PU.1	[87]
UP7	M00658	PU.1	*Sfpi1*	Known regulator of hematopoiesis	[88]
	M00329	Pax-9	*Pax9*		
	M00925	AP-1	*Jun*	Known regulator of myeloid development	[89]
	M01031	HNF4	*Hnf4a*		
	MA0081	SPI-B	*Spib*	Can functionally replace PU.1 in myeloid development	[85]
UP01	M00217	USF	*Usf1*	Regulates HOXB4 in normal and leukemia stem cells	[90]
	M00792	SMAD	*Smad1*		
	M00805	LEF1	*Lef1*	Expression altered in acute leukemia	[77]
	M00799	Myc	*Myc*	Upregulated in AML and induces AML	[91]
	MA0002	AML-1	*Runx1*	Known regulator of hematopoiesis	[88]
UP67	M00133	Tst-1	*Pou3f1*		
	M00188	AP-1	*Jun*	Known regulator of myeloid development	[89]
	M00729	Cdx-2	*Cdx2*	Involved in the ETV6-CDX2 fusion protein	[92]
	M00912	C/EBP	*Cebpa*	Known regulator of hematopoiesis	[88]
	M00162	Oct-1	*Pou2f1*	Regulates PU.1	[87]

PAP was used to identify overrepresented TF binding sites in each coexpressed gene clusrer. Almost all of these TFs are associated with myeloid development or myeloid leukemia.

### Myeloid genetic networks among transcription factor genes

**Figure 4:**
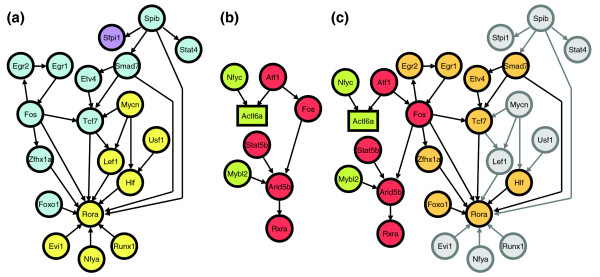
Genetic networks operating in myeloid development and APL. In these networks, circle nodes represent TF genes. Genes that do not encode TFs are shown in rectangles. An arrow is drawn from TF-A to gene-B if TF-A regulates gene-B. **(a) **The predicted genetic network operating in myeloid differentiation. Multiple regulatory pathways in this network converge on one single TF, Rora. The expression profiles of the TF genes are color coded: blue, upregulated at day 0; yellow, upregulated at day 0 and day 1; purple, upregulated at day 7. **(b) **The seven TFs that are dysregulated in APL may be connected to form a common regulatory pathway. Aberrant expressions of these TFs are color coded: red, overexpression; green, underexpression. **(c) **The perturbation of the normal network by dysregulated TFs in APL. The normal and disease regulatory pathways converge on Rora and Rxra, respectively. The dysregulated pathway in APL may perturb the normal genetic network through Fos. Furthermore, many TFs in the normal network (shown in orange nodes) are predicted as direct targets of at least one TF dysregulated in APL (Table 4).

To systematically study the transcriptional regulatory mechanisms underlying myeloid differentiation, focus was given to TF genes in each coexpressed gene cluster. These TFs presumably modulate myeloid development by regulating the genes in the same gene cluster (that is, genes that were coexpressed with the TFs). Indeed, 12 of the 17 TFs found in these gene clusters were previously associated with myeloid differentiation or myeloid disorders (Table 2). Therefore, the regulatory networks of TFs were extracted from each genetic network identified for each gene cluster (Additional data file 8). Because genes in each coexpressed cluster are upregulated at different time points during myeloid development, each individual network represents a 'sub-network' of the entire transcriptional regulatory network for myeloid differentiation. Therefore, a comprehensive transcriptional regulatory network was constructed by combining each individual network identified in each coexpressed gene cluster. Namely, each individual network was joined by the common TFs to build a combined network (Figure 4a).

**Table 2:** Transcription factor genes identified in coexpressed gene clusters

Cluster	Gene ID	Symbol	Expression correlation coefficient	Association with myeloid development	Reference
UP0	13653	*Egr1*	0.9928	Stimulates development of hematopoietic progenitor cells	[35]
	13654	*Egr2*	0.9459		
	14013	*Evi1*	0.9373	Involved in many myeloid disorders	[76]
	14281	*Fos*	0.9488	Modulates myeloid cell survival and differentiation	[42]
	16842	*Lef1*	0.9325	Expression altered in acute leukemia	[77]
	17131	*Smad7*	0.9041	Alters cell fate decisions of human hematopoietic repopulating cells	[78]
	18109	*Mycn*	0.9094		
	19883	*Rora*	0.9335		
	20849	*Stat4*	0.9562	Expressed in early myeloid development	[79]
	21414	*Tcf7*	0.9614		
	56458	*Foxo1*	0.9575	Activates the mixed lineage leukemia gene	[80]
UP01	14013	*Evi1*	0.9042	Involved in many myeloid disorders	[76]
	14391	*Gabpb1*	0.9377	Activates the neutrophil elastase promoter	[81]
	16842	*Lef1*	0.9593	Expression altered in acute leukemia	[77]
	18044	*Nfya*	0.9036		
	18109	*Mycn*	0.9509		
	19883	*Rora*	0.9525		
	217082	*Hlf*	0.9537	E2A-HLF fusion abrogates apoptosis in leukemia cells	[82]
UP67	12013	*Bach1*	0.9274		
	17119	*Mxd1*	0.9168	Expression induced during myeloid development	[83]
	328572	*Ep300*	0.9097	Regulates Runx1 through acetylation of lysine residues	[84]

These TFs were all upregulated on just one day or over two consecutive days during the seven-day *in vitro *myeloid differentiation. Twelve of these seventeen TF genes aare known to regulate myeloid differentiation or be involved in myeloid leukemia.

The identified genetic network for myeloid differentiation has several interesting features. First, the integration of individual networks into the complete myeloid development network was consistent with the time at which each individual gene cluster was upregulated (that is, genes upregulated earlier occupied the upper part of the network and genes upregulated later occupied the lower part). Second, multiple regulatory pathways converge on a single TF, Rora. These characteristics highlight the genetic circuitry that may be operating in the myeloid differentiation.

### Genetic networks in acute promyelocytic leukemia

To identify transcriptional regulatory mechanisms that are dysregulated in acute promyelocytic leukemia, TFs that are differentially expressed in APL compared to normal cells were identified as follows. Gene expression profiles in APL were collected from a mCG-PML-RARα knock-in mouse model [30]. PML-RARα is a fusion protein observed in most APL patients, and the majority of PML-RARα knock-in mice eventually develop APL [30]. Because APL is characterized by an arrest of the normal myeloid differentiation program in the promyelocytic stage, and promyelocytes are the predominant cells at day 2 and day 3 of the *in vitro *myeloid maturation program, gene expression data measured at these two days were compared to those measured in APL mice. The software program SAM (Significance Analysis of Microarrays) [40] was used to identify 602 differentially expressed genes in APL. Among these genes, 472 were overexpressed in APL, and 130 were underexpressed. These differentially expressed genes include seven encoding TFs (Stat5b, Fos, Atf1, Arid5b, Rxra, Mybl2, Nfyc) that have characterized binding profiles, termed the APL dysregulome (Table 3). Four of the seven dysregulated TFs, Atf1, Fos, Rxra and Stat5b, have been previously associated with acute myeloid leukemia [41-44], and ninety-six genes that were differentially expressed in APL were identified as targets of these TFs (Additional data file 9).

**Table 3:** Dysregulated transcription factors in APL

Gene ID	Symbol	Gene name	APL expression	SAM score
20851	*Stat5b*	signal transducer and activator of transcription 5B	Up	12.05
14281	*Fos*	FBJ osteosarcoma oncogene	Up	9.71
11908	*Atf1*	activating transcription factor 1	Up	9.68
71371	*Arid5b*	AT rich interactive domain 5B (Mrf1 like)	Up	9.63
20181	*Rxra*	retinoid X receptor alpha	Up	9.48
17865	*Mybl2*	myeloblastosis oncogene-like 2	Down	-9.47
18046	*Nfyc*	nuclear transcription factor-Y gamma	Down	-10.21

SAM was used to identify seven TFs that are differentially expressed in APL and in normal promyelocytes. Five of these TFs are upregulated in APL, while two of them are downregulated.

To test if these seven TFs in the APL dysregulome participate in a common regulatory pathway (that is, their abnormal expression is the cause or result of a single regulatory cascade), transcriptional regulatory interactions between these TFs were identified using regulatory sequence analysis (see above and Materials and methods). Namely, TF-A and TF-B are connected to form a regulatory pathway if TF-B has over-represented binding sites of TF-A. As a result, six of these seven TFs were shown to form a common regulatory pathway (Figure 4b). The last TF, Nfyc, neither regulates nor is regulated by any of the other six TFs, but it regulates a gene (*Actl6a*) in common with Atf1. Two of these seven TFs, Nfyc and Mybl2, are expressed at lower levels in APL than in normal promyelocytes. It is interesting to note that while Atf1 regulates Fos and Actl6a, Fos is up-regulated and Actl6a is down-regulated in APL. This suggests that Atf1 may act as both a transcriptional activator and a repressor, possibly depending on different cooperative factors. This hypothesis is supported by a previous study of Atf1 [45]. Thus, to identify cooperative TFs of Atf1, TFs that regulate Fos or Actl6a and have similar expression profiles to Atf1 in APL were identified. This analysis identified Egr2 and Nfyb as cofactors of Atf1 in the regulation of Fos and Actl6a, respectively.

To study how the APL dysregulome perturbs the genetic network of normal myeloid differentiation, the regulatory cascade of the seven dysregulated TFs were joined with the normal myeloid genetic network (Figure 4c). All the predicted regulatory interactions between any two TFs within the normal genetic network were maintained, and the TFs that were predicted as targets of at least one dysregulated TF were identified (Table 4, Figure 4c). Interestingly, these two networks could be simply combined through a common TF, Fos. This result suggests that the genetic network of normal myeloid differentiation is perturbed, and this dysregulation is mediated through Fos. Furthermore, these results predict a change in the genetic circuitry wherein the normal cascade is regulated by Rora while the pathophysiology observed in APL is mediated by Rxra. *Bona fide *downstream targets of Rora and Rxra need to be identified, and their functions in normal myeloid development or APL need to be elucidated to further validate the role of Rora and Rxra in normal or leukemic biology.

**Table 4:** Transcription factors in the normal myeloid genetic network regulated by dysregulated transcription factors in APL

Differentially expressed TFs in APL	Regulated TFs in normal myeloid development	*P *value
Arid5b	Rora	~0
Arid5b	Tcf7	0.0019
Atf1	Egr1	~0
Atf1	Hlf	0.0041
Atf1	Rora	0.0015
Atf1	Smad7	0.0015
Fos	Egr2	0.0005
Fos	Rora	~0
Fos	Tcf7	0.0036
Fos	Zfhx1a	0.0005
Mybl2	Rora	~0
Rxra	Egr1	0.0003
Rxra	Etv4	0.0014
Stat5b	Foxo1	0.0005

These regulatory targets were identified using permutation of TF binding sites.

### PML-RARα and APL pathogenesis

While the proposed genetic network predicted that the APL pathway converged on Rxra, the relationship between PML-RARα and the APL dysregulome was not uncovered. Thus, to test whether the APL dysregulome (Figure 4b) is caused either directly or indirectly by PML-RARα, PML-RARα TF targets were identified using RARα binding profiles. Because it has been shown that PML-RARα binds to a much broader range of binding site architectures than the normal RARα, eight binding profiles of PML-RARα with various orientations and spacings (DR2, DR3, DR4, DR5, DR6, DR12, IR0, and ER8) were created based on a previous experimental study [46] in addition to the RARα binding profiles in TRANSFAC. Using the same binding site permutation algorithm, none of the TFs in the APL dysregulome were predicted as direct targets of PML-RARα, suggesting that dysregulation of these TFs was mediated by other TFs.

**Figure 5:**
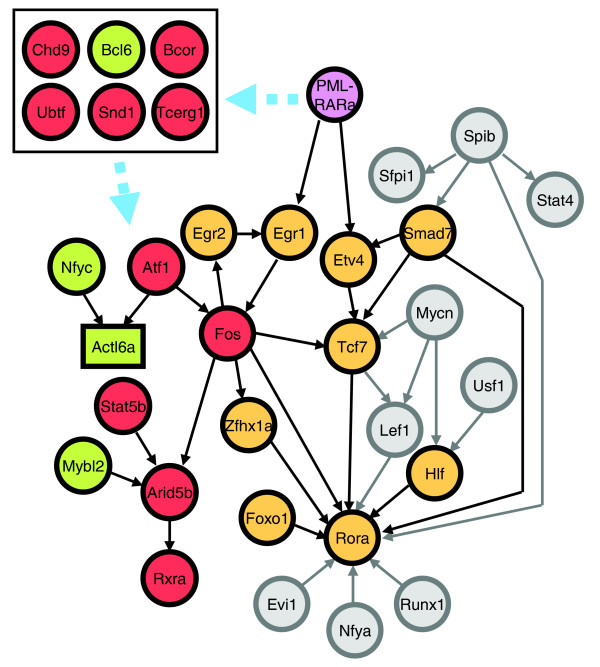
The proposed model of APL pathogenesis induced by PML-RARα. PML-RARα may activate the dysregulation of several TFs in the disease regulatory pathway in APL through six mediator TFs (dashed blue arrow). This regulatory circuitry ultimately converges on the overexpression of Rxra. Red circle, overexpressed TFs; green circle, underexpressed TFs; green box, underexpressed genes; orange circle, TFs in the normal network that are predicted as targets of dysregulated TFs in APL; gray circle, other TFs in the normal network; purple circle, PML-RARα.

Thus, to identify direct targets of PML-RARα, gene expression profiles collected from cultured bone marrow cells derived from young mCG-PML-RARα knock-in mice were used (see Materials and methods). First, to identify genes dysregulated in preleukemic promyelocytes, expression data from days 0, 2, and 7 in normal and preleukemic promyelocytes were analyzed using SAM. No genes were differentially expressed at day 0, whereas 73 and 1,028 genes were differentially expressed at day 2 and day 7, respectively. Using the eight binding profiles of PML-RARα and the RARα binding profiles in TRANSFAC, six TFs differentially expressed at day 7 were predicted as direct PML-RARα targets (Table 5). Interestingly, one of the TFs in the normal network, Egr1, is also predicted as a PML-RARα target; however, Egr1 is expressed at a normal level in young, preleukemic mice. Therefore, it is possible that Egr1 may not be a direct mediator of PML-RARα in leukemogenesis. Collectively, these results suggest a model of APL pathogenesis in which PML-RARα regulates the APL dysregulome through six mediator TFs. This circuitry ultimately converges to create the APL dysregulome, hallmarked by activation of Rxra, which then triggers downstream events (Figure 5).

**Table 5:** Predicted mediator transcription factors of PML-RARα

Gene ID	Symbol	Gene name	APL expression	SAM score
12053	*Bcl6*	B-cell leukemia/lymphoma 6	Down	-7.39
21429	*Ubtf*	upstream binding transcription factor, RNA polymerase I	Up	4.49
56070	*Tcerg1*	transcription elongation regulator 1 (CA150)	Up	6.25
56463	*Snd1*	expressed sequence AL033314	Up	4.44
71458	*Bcor*	Bcl6 interacting corepressor	Up	4.90
109151	*Chd9*	chromodomain helicase DNA binding protein 9	Up	5.64

SAM and the binding profiles of Rara and PML-RARα were used to identify direct regulatory targets of PML-RARα. These six TFs may activate the dysregulation of other aberrantly expressed TFs in APL.

## Discussion

### A novel approach to genetic network identification

In this report, we propose a novel approach to genetic network identification that combines two independent types of information, gene expression profiling data and computational identification of TF binding sites. Using gene expression data, genes coherently expressed with TFs were first identified. In parallel, direct regulatory targets of TFs were predicted by a computational model that calculates binding scores for each coexpressed gene and assesses statistical significance using binding site permutation. These two types of information were then integrated to construct the genetic network for each coexpressed gene cluster, which were subsequently consolidated into a comprehensive network. We used this approach to identify the genetic network in normal myeloid differentiation and to determine how this network is perturbed in APL. This approach is general and may be applied to delineate genetic networks operating in other complex human diseases.

### Prediction of direct regulatory targets using TF binding site identification

A major challenge in expression data-based genetic network construction is the ability to distinguish direct regulatory targets of TFs from indirectly regulated downstream genes. To predict direct targets accurately, coexpressed genes were scored using the identified TF binding sites, and the statistical significance of each score was determined. This method is different from other existing approaches in several ways. First, it does not compare the frequency of TF binding sites to a specific set of reference sequences. Instead, the binding scores of genes in the entire genome are calculated, and the *P *value for observing a score is determined by permutation of all the binding sites. Secondly, unlike most previous methods where TF binding sites are only identified in the proximal promoter region, our model considers binding sites located in evolutionarily conserved sequences in the entire gene locus. This includes a significant number of additional, highly conserved sites found in introns and distant genomic regions. These modifications and improvements make our predictions more accurate due to a better scoring model and more comprehensive due to a more complete set of evolutionarily conserved TF binding sites [47].

### Using the *in vitro *cell culture system to model human myeloid differentiation

In this study, we used an *in vitro*, G-SCF driven myeloid differentiation system to model normal myeloid maturation and compared its gene expression profile to that of an *in vivo *APL mouse model. Although the *in vitro *GCS-F driven myeloid differentiation is not equivalent to *in vivo *differentiation, it is a validated surrogate that mimics human myeloid differentiation [30]. Moreover, this system is currently the only practical and technically feasible platform for the study of normal murine myeloid development. Therefore, we believe the data provided by this system can be used to infer hematopoietic gene regulation.

### Comparing the identified genetic networks with previous experimental data

The genetic network of TFs identified by our computational method provides several new insights into the normal and aberrant regulatory pathways that may drive myeloid differentiation and in acute promyelocytic leukemia, respectively. These predictions are compared to several previous findings.

First, we observed that the normal and disease regulatory cascades converge on Rora and Rxra, respectively. Rora and Rxra belong to the same family of nuclear receptors and are related to Rara, and Rxra forms a heterodimer with Rara. Although the specific functions of Rora in myeloid differentiation are still unknown, all three nuclear receptors bind to very similar DNA sequences ((A/G)GGTCA) [46,48], implying that they may regulate the same genes. In addition, the human *RORA *gene is located within a highly conserved region on chromosome 15 near the human *PML *gene [49]. Therefore, our results suggest a novel role for Rora in myeloid development. Furthermore, the dysregulated pathway in APL leads to an overexpression of Rxra, and the absence of Rxra in the normal network implies that Rxra is a potential effector protein in APL but not in normal myeloid development. In fact, recent studies have shown that although Rxra is not required in normal myelopoiesis [50], it is an essential component for the PML-RARα complex to initiate APL in mice [51,52]. Therefore, the prediction of Rxra as a potential key participant in APL pathogenesis is supported by *in vivo *studies.

Second, the normal and the disease pathways did not share many common TFs. Instead, only one TF in the normal network, Fos, which modulates myeloid cell survival and differentiation [42], was dysregulated in APL. Our analysis identified that the TFs dysregulated in APL may mediate the perturbation of the normal myeloid genetic network through Fos. Indeed, previous studies have demonstrated that PML-RARα promotes cell growth by activating Fos [53], and that PML-RARα directly functions as a co-repressor of Fos in the absence of retinoic acid [54]. Our data suggest that over-expression of Fos in APL may have an aberrant activation function as well. This over-expression of Fos may be induced by PML-RARα and may, in turn, cause the dysregulation of other TFs involved in APL.

Third, we identified a set of six TFs as direct targets of PML-RARα. Among these six TFs, five of them were overexpressed in APL, and one TF was underexpressed, suggesting PML-RARα may act as both a repressor and an activator. In agreement with this observation, PML-RARα was first demonstrated as an enhanced repressor of retinoic acid target genes by its stronger binding to corepressors than wild-type RARα [55,56], but it is becoming clear that PML-RARα may also function as an activator [54,57]. While a version of PML-RARα that can only repress gene expression is still leukemogenic [58], whether PML-RARα can trigger APL pathogenesis by gene activation is unknown. Our analysis suggests that PML-RARα may indeed be an activator and provides a potential mechanism through which this may occur.

Finally, our data are consistent with a previous study that showed that relative levels of Sfpi1 regulate macrophage versus neutrophil differentiation [59]. Importantly, at low Sfpi1 levels (neutrophil differentiation), we did not find that *Egr1*/*2 *are targets of Sfpi1 whereas Laslo *et al*. [59] found that *Egr1*/*2 *are targets of Sfpi1 during macrophage differentiation where Sfpi1 is expressed at high levels. Therefore, there may be weaker binding sites for Sfpi1 in *Egr1 *or *Egr2 *promoters, and thus the activation of *Egr1 *and *Egr2 *may require a higher expression level of Sfpi1.

### Genetic networks may be expanded using additional information on binding profiles

The computational approach used in this study is based on the binding profiles of TFs in TRANSFAC and JASPAR. Therefore, the quality of our prediction and the accuracy of our conclusions may be dependent upon the quality of the binding models in these databases. Some TFs require other co-factors to accomplish their regulatory functions, and so their binding specificities or preferences may also be dependent on their binding partners. Such information may not have been included in the TRANSFAC or JASPAR models. For example, the TF RXRA studied in this work typically forms a heterodimer with various co-factors, including RAR, VDR, TR, or PPAR at various spacings. The four binding profiles we used were created using different binding partners of RXRA, including PPAR (M00518), RAR and TR (M00963), and VDR (M00966). Thus, our prediction did consider the binding site of RXRA with different binding partners. However, our prediction may not include all the allowable spacings between the two sites. Therefore, our results may be further improved when more precise and complete binding profiles are available.

While the direct targets of PML-RARα in APL were computationally identified, the direct targets of these mediator TFs could not be determined. Therefore, the genetic network in APL was not fully elucidated (Figure 5). In order to identify targets of these mediator TFs, knowledge of their binding profiles is required, information that is currently not available. In fact, while there are more than 2,000 TFs predicted in the human genome [60], only a quarter of them have known binding profiles. Thus, the predicted genetic network may be further improved and expanded when more TF binding profiles become available. Additional TF binding profiles may be generated by traditional protein-DNA binding assays [61] or by computational approaches that utilize evolutionary conservation of functional sequences. This latter approach is used to predict DNA binding profiles on a genome-wide scale. For example, DNA binding patterns may be identified by calculating the conservation rate of a given oligonucleotide across the genome [62], or by clustering genes that share common conserved sequences [63]. Thus, the employment of such methods is a rational next step toward the refinement of genetic networks. Integration of this computational component would not only increase our understanding of the molecular mechanisms underlying APL but would also facilitate the construction of more comprehensive regulatory networks driving other complex diseases.

## Conclusion

We have developed an integrated approach to mammalian genetic network construction by combining gene expression profiling data and TF binding site identification. Using this technique, we have predicted Rxra as a key regulator in APL and Fos as one of the key mediators of PML-RARα. These results provide new insights about the pathophysiology of APL. Our approach may be applied to study the genetic circuitry operating in other complex diseases in humans.

## Materials and methods

### Genomic sequence collection and ortholog identification

The genomic sequences of human, mouse, and rat were acquired from the NCBI's Genome Assembly Project [64]. Genome build 35 was used for human, build 33 for mouse and build 3 for rat. The genomic sequence of a gene locus was defined as the sequence between the end of the upstream gene and the end of the gene itself. Within this range, protein coding sequences were masked and excluded from the search of TF binding sites. Repetitive elements were also masked by the program RepeatMasker [65] using slow and sensitive mode (the -s flag). Human, mouse and rat ortholog gene groups (13,194 in total) were identified using the annotation of NCBI's HomoloGene database as previously described [29]. Genomic sequences of the genes in the same ortholog group were then aligned using the program TBA [66].

### TF binding site identification and binding probability score calculation

To identify TF binding sites, 596 vertebrate TF binding profiles were collected from the TRANSFAC (version 9.1) and JASPAR databases. The program PATSER [67] was used to search for matches of these profiles in the genome using default cutoff scores (the -li option). This cutoff score is calculated as follows: for each position, PATSER scores the subsequence and calculatesthe *P *value for observing the same score or higher at thatposition [68]. A *P *value cutoff is calculated for each binding profile using its information content. The score corresponding to that *P *value cutoff is then chosen to be the cutoff score. After all the TF binding sites were identified, binding probability scores [29] for each TF-gene pair were then calculated using evolutionarily conserved TF binding sites found in the non-coding sequence of a gene. For mammals, functional regulatory elements have been found in distant upstream regions [69,70] as well as intronic sequences [71,72]. However, searching for TF binding sites in the entire intergenic sequence and in the entire gene locus frequently results in a high false discovery rate. Therefore, to overcome this problem, we considered only evolutionarily conserved TF binding sites in the 'proximal promoter region' of a gene and in the most conserved sequence regions within a gene locus defined by multi-species conserved sequences (MCSs) [73].

The proximal promoter region of a gene was defined as the 10 kb upstream sequence and the 5 kb downstream sequence from the transcription start site, regardless of the presence of an upstream gene or a downstream gene. MCSs are defined as the top 5% conserved sequences in the human genome when compared to another 11 vertebrate genomes [73]. The human MCSs were first downloaded, and the MCSs in mouse and rat were defined by mapping the human MCSs to the mouse or rat genomes using multiple sequence alignments generated by TBA. These multiple sequence alignments were also used to identify evolutionarily conserved TF binding sites, which were defined as sites located within conserved sequence regions, present in all the species and aligned in the multiple sequence alignment [74].

### Calculating statistical significance for a binding probability score

To predict regulatory targets of TFs, genes that have statistically significant binding scores [29] for a TF were identified. The statistical significance of a given binding score was evaluated by the *P *value for observing an equal or higher score by chance. This *P *value was calculated by permutation of all the TF binding sites in the genome. In this algorithm, each individual binding site of a TF was randomly assigned to genes in the genome based on a precalculated probability distribution calculated as follows: the probability for a gene to acquire a given TF site is the length of the TF binding site search range of that gene (that is, the proximal promoter region and the MCSs; see above) divided by the sum of the TF binding site search ranges of all the genes in the genome. This permutation of TF binding sites was performed for 10,000 iterations. After each iteration, a new binding probability score was calculated for each gene using the TF sites randomly assigned to that gene. The *P *value for observing a score for a gene was then calculated by the number of iterations where a binding score equal to or higher than the true score was obtained for that gene, divided by the total number of iterations.

### Gene expression profiling data

Gene expression profiles in normal myeloid differentiation were obtained from a G-CSF stimulated *in vitro *myeloid differentiation model [30]. Expression data were collected from cultured bone marrow cells in two independent experiments during a seven-day time course. Gene expression data in APL were collected from bone marrow cells of six adult PML-RARα knock-in mice based on a previously developed murine APL model [30]. Expression profiles of the cultured bone marrow cells were also collected from two sets of young PML-RARα knock-in mice. For the expression data collected from cultured cells, only probesets that were present on at least one day in at least one experiment were considered.

### Coexpressed gene cluster identification

The coexpressed gene clusters during myeloid differentiation were identified using the template matching clustering tool in the software suite FunctionExpress [75]. The templates for each upregulated expression pattern were created manually, including genes that were upregulated on just one day or over two consecutive days during the seven day time course. The Pearson's correlation coefficient was used to quantify the similarity between the template and the expression profile of each probeset. A cutoff of 0.9 was applied to the correlation coefficient to identify the coexpressed gene clusters.

### Additional myeloid TF identification

PAP [29] was used to identify additional TFs that may regulate genes in each coexpressed gene cluster. For each gene cluster, PAP ranked all the TFs by their R-scores, which were calculated based on overrepresentation of their binding sites in the coexpressed genes. To find TFs that have a statistically significant R-score, a *P *value for each R-score was calculated using 10,000 randomly selected gene clusters of the same size. For each random set, the R-scores for each TF were calculated, and the *P *value for a TF was calculated as the number of gene clusters that had an equal or higher score than that of the original gene cluster, divided by 10,000, the total number of random sets.

## Abbreviations

AML, acute myeloid leukemia; APL, acute promyelocytic leukemia; G-CSF, granulocyte colony-stimulating factor; MCSs, multi-species conserved sequences; SAM, Significance Analysis of Microarrays; TF, transcription factor.

## Authors' contributions

LWC, RN and GDS developed the approach and wrote the manuscript. LWC performed the analysis. JEP, WY and TJL provided the experimental data. JEP and TJL participated in the discussion of the results and edited the manuscript.

## Additional data files

The following Additional data files are available with the online version of this paper. Additional data file 1 is a spreadsheet showing the number of genes that were up-regulated at any number of consecutive days during the *in vitro *myeloid development system. Additional data file 2 is a spreadsheet containing a list of genes identified in each coexpressed gene cluster. Additional data file 3 is a spreadsheet containing a list of predicted regulatory targets of TFs identified in each coexpressed gene cluster. Additional data file 4 is a spreadsheet containing a list of down-regulated genes in the *in vitro *myeloid differentiation system. Additional data file 5 is a PDF file showing the complete genetic networks (including genes that do not encode TFs) for genes upregulated at day 0, and at day 0 and day 1. Additional data file 6 is a PDF file showing the expanded genetic networks (including additional TFs identified by PAP) for genes upregulated at day 0, and at day 0 and day 1. Additional data file 7 is a spreadsheet containing a list of myeloid TFs identified by PAP and their regulatory relationships to other genes in the myeloid development networks. Additional data file 8 is a PDF file showing regulatory networks for TF genes identified in each coexpressed gene cluster. Additional data file 9 is a spreadsheet containing a list of TFs dysregulated in APL and their predicted regulatory targets.

## Supplementary Material

Additional data file 1 These genes were identified by calculating the Pearson's correlation coefficients of their expression profiles to each individual expression pattern. Click here for file

Additional data file 2 Genes identified in each coexpressed gene cluster. Click here for file

Additional data file 3 Predicted regulatory targets of TFs identified in each coexpressed gene cluster. Click here for file

Additional data file 4 Down-regulated genes in the *in vitro *myeloid differentiation system. Click here for file

Additional data file 5 **(a) **Genes upregulated at day 0; **(b) **genes upregulated at day 0 and day 1. Click here for file

Additional data file 6 **(a) **Genes upregulated at day 0; **(b) **genes upregulated at day 0 and day 1. Additional TFs identified by PAP and their regulatory relationships to other genes in the myeloid networks are colored red. Click here for file

Additional data file 7 Myeloid TFs identified by PAP and their regulatory relationships to other genes in the myeloid development networks. Click here for file

Additional data file 8 **(a) **TF genes upregulated at day 0. **(b) **TF genes upregulated at day 0 and day 1. **(c) **TF genes upregulated at day 6 and day 7. **(d) **TF genes upregulated at day 7. Color coding in these networks denotes how these TF genes were identified: blue, TFs identified by coexpression; green, additional TFs identified by PAP; yellow, TFs identified by both coexpression and PAP. Click here for file

Additional data file 9 TFs dysregulated in APL and their predicted regulatory targets. Click here for file
