# Supplementary figures and images for: Computational identification of the normal and perturbed genetic networks involved in myeloid differentiation and acute promyelocytic leukemia
Source: Genome Biol. 2008 Feb 21;9(2):R38. doi: 10.1186/gb-2008-9-2-r38 (PMC2374711; doi:10.1186/gb-2008-9-2-r38)

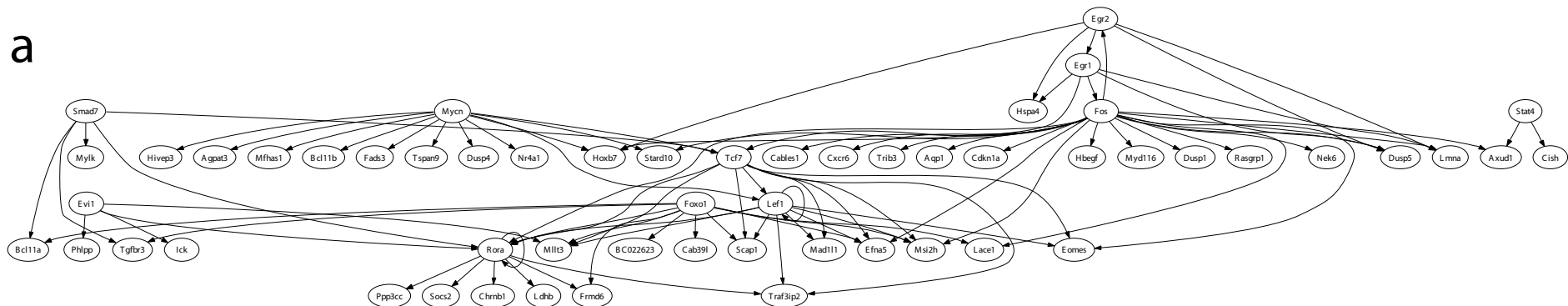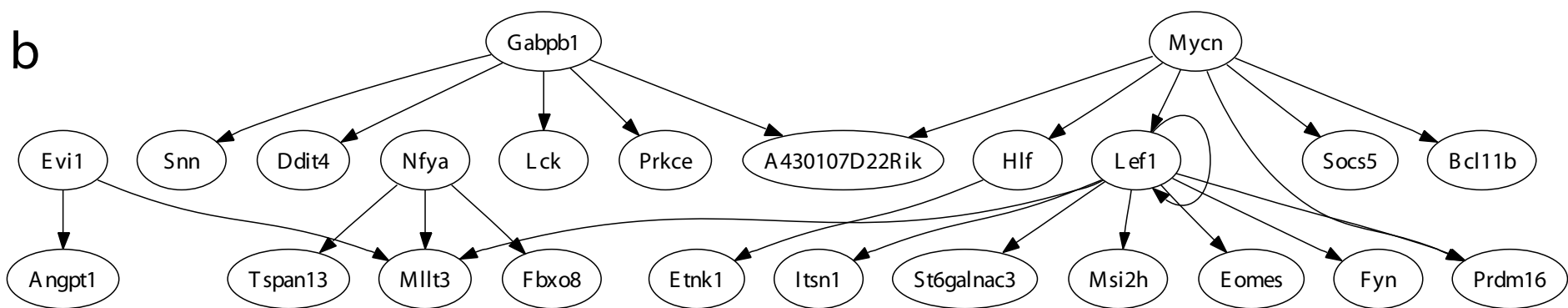

Supplement: Additional data file 5 — (a) Genes upregulated at day 0; (b) genes upregulated at day 0 and day 1. [file gb-2008-9-2-r38-S5.pdf]

a

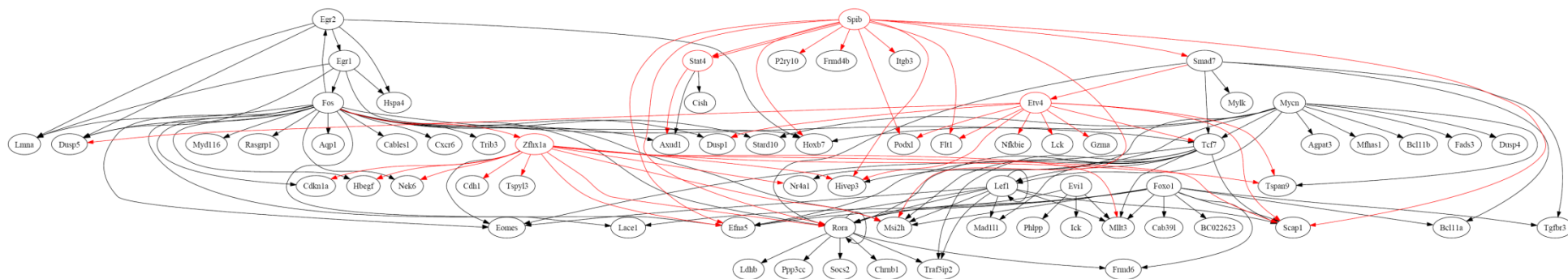

b

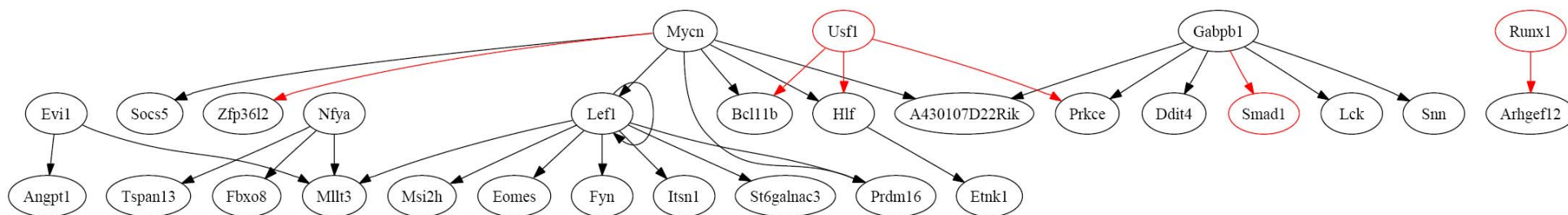

Supplement: Additional data file 6 — (a) Genes upregulated at day 0; (b) genes upregulated at day 0 and day 1. Additional TFs identified by PAP and their regulatory relationships to other genes in the myeloid networks are colored red. [file gb-2008-9-2-r38-S6.pdf]

**a**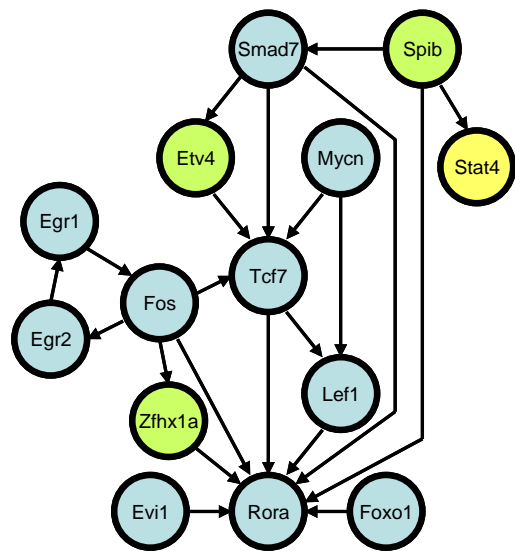**b**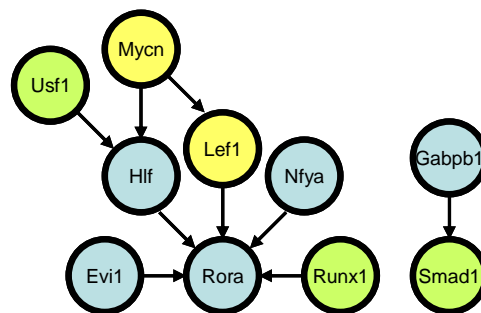**c**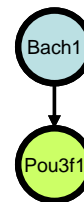**d**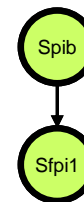

Supplement: Additional data file 8 — (a) TF genes upregulated at day 0. (b) TF genes upregulated at day 0 and day 1. (c) TF genes upregulated at day 6 and day 7. (d) TF genes upregulated at day 7. Color coding in these networks denotes how these TF genes were identified: blue, TFs identified by coexpression; green, additional TFs identified by PAP; yellow, TFs identified by both coexpression and PAP. [file gb-2008-9-2-r38-S8.pdf]
